# Value Learning and Arousal in the Extinction of Probabilistic Rewards: The Role of Dopamine in a Modified Temporal Difference Model

**DOI:** 10.1371/journal.pone.0089494

**Published:** 2014-02-26

**Authors:** Minryung R. Song, Jean-Marc Fellous

**Affiliations:** 1 Department of Bio and Brain Engineering, Korea Advanced Institute of Science and Technology (KAIST), Daejeon, South Korea; 2 Graduate Interdisciplinary Program in Neuroscience, University of Arizona, Tucson, Arizona, United States of America; 3 Department of Psychology, University of Arizona, Tucson, Arizona, United States of America; 4 Department of Applied Mathematics, University of Arizona, Tucson, Arizona, United States of America; Duke University Medical Center, United States of America

## Abstract

Because most rewarding events are probabilistic and changing, the extinction of probabilistic rewards is important for survival. It has been proposed that the extinction of probabilistic rewards depends on arousal and the amount of learning of reward values. Midbrain dopamine neurons were suggested to play a role in both arousal and learning reward values. Despite extensive research on modeling dopaminergic activity in reward learning (e.g. temporal difference models), few studies have been done on modeling its role in arousal. Although temporal difference models capture key characteristics of dopaminergic activity during the extinction of deterministic rewards, they have been less successful at simulating the extinction of probabilistic rewards. By adding an arousal signal to a temporal difference model, we were able to simulate the extinction of probabilistic rewards and its dependence on the amount of learning. Our simulations propose that arousal allows the probability of reward to have lasting effects on the updating of reward value, which slows the extinction of low probability rewards. Using this model, we predicted that, by signaling the prediction error, dopamine determines the learned reward value that has to be extinguished during extinction and participates in regulating the size of the arousal signal that controls the learning rate. These predictions were supported by pharmacological experiments in rats.

## Introduction

If the conditioned stimulus (CS) is no longer followed by the unconditioned stimulus (US), it weakens the ability of the CS to induce the conditioned response, a phenomenon called extinction [1], [2]. Extinction is not erasing prior beliefs about the contingency of the CS and US but amounts to adding a new belief that the CS does not elicit the US anymore. This is evidenced by the finding that the extinguished conditioned response typically reappears as time goes by (spontaneous recovery). Unlike *de novo* acquisition of the association between the CS and US, extinction occurs under the influence of prior beliefs about the CS-US relationship and usually takes longer than acquisition. It has been suggested that arousal induced by observations inconsistent with prior beliefs facilitates the formation of a new belief by enhancing the learning rate [3], [4], [5].

In the stochastic and continuously changing environment, the extinction of probabilistic rewards is important for survival and failure in the extinction of probabilistic rewards leads to maladaptation such as pathological gambling [6], [7]. In cases of the extinction of probabilistic rewards, the amount of arousal elicited by the cessation of those rewards increases with the reward probability. Research has shown that after extensive learning of the values of probabilistic rewards, the lower the reward probability, the more time is required for extinction to occur (a phenomenon known as partial reinforcement extinction effect (PREE)) [8], [9]. The PREE can be explained by an arousal-mediated difference in the learning rate of the extinction of low and high probability rewards. Furthermore, if the extinction happens after a moderate amount of learning, the rate of extinction appears as an inverted-U shaped function of the reward probability [9], [10]. This indicates that the learned reward value as well as arousal may play a role in the extinction of probabilistic rewards.

The involvement of reward learning and arousal elicited by deviations from prior beliefs suggests that the midbrain dopaminergic system may be a neural substrate that controls the rate of the extinction of probabilistic rewards. Dopamine neurons are known to drive reinforcement learning by signaling the reward prediction error–discrepancy between the expected reward and the reward received [11], [12]. The magnitude of the phasic firing of dopamine neurons at the time of the CS is related to the parameters of the rewards including their probability, size and delivery delay [13], [14]. Because dopamine neurons signal the reward prediction error, they are in a good position to not only affect reward learning but also to signal deviations from prior beliefs. Recent studies have demonstrated that the activity of the amygdala reflected the level of arousal resulting from deviations from prior beliefs [15], [16] and that enhanced levels of arousal are related to larger learning rate [4]. The arousal signal in the amygdala has also been found to rely on the dopamine neurons [17]. Finally, systemic injection of amphetamine has been found to abolish PREE, supporting again a potential role of the dopamine neurons in PREE [18].

During extinction, the phasic activity of dopamine neurons in response to the CS decays and an inhibitory response to the CS develops [19], [20]. The temporal difference model by Pan et al. [20] is a biologically grounded model based on these findings and simulates key features of reward extinction such as spontaneous recovery and fast relearning after extinction. However, this model has not been tested in the extinction of probabilistic rewards and does not take into account the effect of arousal. The Pearce-Kaye-Hall model proposes a way to incorporate the prediction error of dopamine neurons and the influence of arousal on the learning rate in reinforcement learning [5], [15]. In this model, the prediction error updates the level of arousal, which correlates with the learning rate.

We hypothesize that the rate of extinction of probabilistic rewards depends on arousal and learned reward value and that the prediction error computed by the dopamine neurons is involved in arousal as well as reward learning. By combining a Pearce-Kaye-Hall model of arousal with the model by Pan et al. [20] we could reproduce the inverted-U shape of the rate of extinction as a function of reward probability after non-extensive learning and could produce PREE after extensive learning. This modified model furthermore predicted the effect of pharmacological activation and inactivation of the dopamine neurons in the ventral tegmental area (VTA) on the extinction of probabilistic rewards. This prediction was confirmed experimentally.

## Materials and Methods

### Subjects

For the behavioral experiments, eight BN/RijHsd and two FBNF1/Hsd rats were used. For the VTA inactivation experiment, six Sprague Dawley and four BN/RijHsd rats were used. For the VTA activation experiment, six BN/RijHsd, two FBNF1/Hsd and three Sprague Dawley animals were used. All rats were male between 5 and 11 months old (Harlan Sprague Dawley). The animals were housed in a reversed 12 h light/dark cycle and maintained at 85% of their normal weight during all experiments. The experiments were approved by the University of Arizona Institutional Animal Care and Use Committee (IACUC; Protocol Number: 06-022). All procedures were conducted in accordance with the Guide for the Care and Use of Laboratory Animals of the University of Arizona IACUC.

### Apparatus and Pretraining

A Plexiglas cylinder (height: 12.5″; diameter: 12″) with three holes (4″ between two adjacent holes) 1.5 inches from the bottom was used. A food pellet (Research Diet, 20 mg) was inserted through the holes with a familiar pair of tweezers. Animals were pre-trained until they ate the food pellets within 15 seconds of tweezer presentation for more than 30 consecutive trials. Inter-trial interval was 20±3 seconds. All pre-training rewards were delivered with 100% probability.

### Experimental Paradigm

We conducted two types of experiments: a trial-constant experiment and a reward-constant experiment. Each experiment was composed of four sessions with different probabilities: 25%, 50%, 75%, and 100% (order counter-balanced). Each session consisted of an acquisition phase and an extinction phase (Table 1). During the acquisition phase of the trial-constant experiment, we fixed the total number of trials to 20 and presented food pellets and empty tweezers in a pseudorandom order according to the designated probability of the session. In contrast, during the acquisition phase of the reward-constant experiment, the number of pellets was fixed to 20 and the total number of trials varied according to the probability of the session. The extinction phase immediately followed the acquisition phase. During the extinction phase, only empty tweezers were presented until the rat did not bite or smell the tweezers for five consecutive trials. The tweezers were inserted every 20 seconds and withdrawn when the animal bit or smelled the tweezers or after 15 seconds if the animal did not (time out). The animals were put back in their home cage for at least 15 minutes between sessions. Each rat underwent both trial-constant and reward-constant experiments (order counter-balanced) on different days.

**Table 1:** Structure of the acquisition phase of the trial-constant and reward-constant experiments.

	Acquisition phase	
Session Probability	Trial-constant	Reward-constant
25%	20 (5)	80 (20)
50%	20 (10)	40 (20)
75%	20 (15)	27 (20)
100%	20 (20)	20 (20)

The numbers outside the parentheses represent the total number of trials whereas those inside represent the number of rewarded trials. The extinction phase immediately followed the acquisition phase in both types of experiments. During the extinction phase, empty tweezers were inserted until the rat made no attempt for 5 consecutive trials.

### VTA Activation/Inactivation Experiments

After pre-training, two stainless steel cannula guides (26 gauge; Plastics One) were implanted bilaterally targeting the VTA (anteroposterior −5.4 mm from bregma; mediolateral ±0.05 mm; dorsoventral −7.5 mm from the surface of the skull). The rats were anesthetized with isoflurane, and their body temperature maintained at 37°C using an isopad during the surgery. Screws and dental acrylic were used to fix the cannula guides to the skull. After one week of recovery, the animals were briefly pre-trained again to confirm that they could perform the task. In the VTA activation experiment group, WIN-255212-2 mesylate 0.5 mM (dissolved in DMSO:Tween:saline = 1∶1∶38; Sigma) 1.4∼1.8 µL or the same amount of physiological saline were microinjected into the VTA each day. WIN-55212-2 is a cannabinoid CB1 receptor agonist which is known to enhance dopaminergic activity [21]. The doses were individually adjusted to the maximum amount of each drug that would not induce abnormal locomotor activity. Saline was used as control. In VTA inactivation experiments, 0.8 µL of bupivacaine hydrochloride 76.9 mM (dissolved in saline; Sigma) or physiological saline were administered instead. The two injections on the same day were followed by different probability sessions separated by a gap of at least 60 min. The order of probability and the order of the drugs were counterbalanced. The trial constant experimental protocol was conducted similarly. All drugs were administered through the injection cannula (33 gauge; Plastics One, about 0.3 µl/min) extending 1 mm beyond the tip of the guide cannula using microliter syringes (Hamilton). The experiment started 2∼5 min after the end of the drug administration.

### Histology

**Figure 1:**
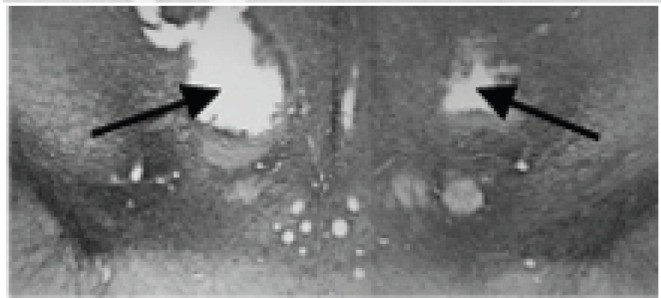
Microphotograph of a representative cannula placement in the VTA (arrows). Cells with tyrosine hydroxylase are stained brown using 3,3′-Diaminobenzidine (DAB).

After all VTA inactivation/activation experiments, the animals were transcardially perfused with 0.9% saline and then 0.4% paraformaldehyde while under deep anesthesia. The brains were first stored in the 0.4% paraformaldehyde solution for 2–6 hours and then transferred to a 30% sucrose solution for at least 24 hours. The brains were cut into 50 µm slices on a cryostat. For tyrosine hydroxylase (TH) staining, free-floating sections were immersed in 3% normal goat serum, 0.02% sodium azide and 0.4% triton X-100 in PBS for 1 hour and then incubated with the primary antibody (rabbit polyclonal anti-TH, 1∶10,000, Chemicon) for 18 hours. The sections were incubated with the biotynilated secondary antibody (1∶1000, in 1.5% NGS, 0.4% Triton X-100 in PBS) for 1 hour and then with ABC complex (1∶500, Vector Lab) for 1 hour. Sections were incubated in 0.05% diaminobenzidine (DAB), 0.003% H_2_O_2_ and 0.05% nickel chloride in PBS (Fig. 1).

### Data Analysis

Reaction times were defined as the time interval between the tweezers presentation and the animals’ attempt to get food from the tweezers–bite or smell. To decrease the effect of individual differences, the average of the first three reaction times for each rat in each session was divided by the average of the first three reaction times of the same rat in all four sessions. The last three reaction times were normalized with the average of the last three reaction times in a similar way. The number of trials until extinction for each rat in each session was divided by the average number of trials until extinction of the same rat in all four sessions. In the VTA activation/inactivation experiments, each rat underwent each probability session under each drug condition up to twice. To normalize these data, the average number of trials until extinction of each rat at each probability under each drug condition was divided by the average number of trials until extinction of the same rat in all four sessions under the saline condition. The reaction times during the acquisition phase and the extinction phase were normalized by the individual average of both drug conditions (saline and WIN-255212-2 or saline and bupivacaine). Normalized data were then analyzed with two-way repeated measures analysis of variance (RMANOVA) and Fishers least significant difference (LSD) method. Differences were assessed significant when p<0.05.

### Model Structure

The computational model was based on that of Pan et al. [20] with the following modifications. First we added an arousal signal. Arousal A(i) in the i-th trial was defined as Li et al. [15]:
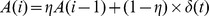
where 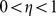 is the decay factor for arousal. δ(t) is the prediction error. During acquisition and extinction, the positive and negative weights were updated as:



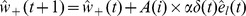





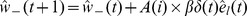
where 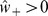 and 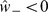 are the positive and negative weight vector, respectively. 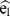 is the eligibility trace. α and β are learning rates for positive and negative weights, respectively. Unlike Pan et al. (2008), we made forgetting occur only during the break since spontaneous recovery occurs over a much longer time scale than acquisition or extinction:



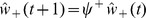





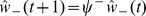
where 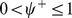 and 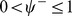 are the decay factors for the positive and negative weight vectors, respectively.

**Figure 2:**
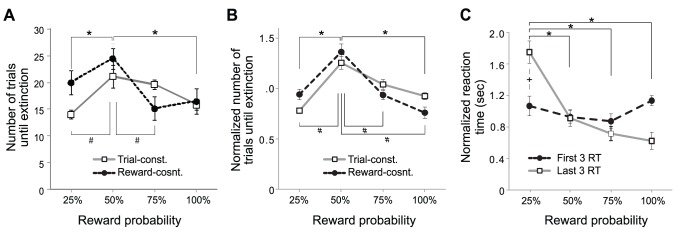
Effects of the reward probability on the rate of extinction. **A:** Number of trials until extinction in trial-constant and reward-constant experiments (* and #: p<0.05 in the trial- and reward- constant experiments, respectively). **B:** Normalized number of trials until extinction in trial-constant and reward-constant experiments (* and #: p<0.05 in the trial- and reward- constant experiments, respectively). **C:** First three and last three reaction times during the acquisition phase of the trial constant experiment (* and +: p<0.05 within the last three reaction times and between the first and last three reaction times, respectively).



A(i) was fixed to 1 for all probabilities during acquisition (see discussion). To simulate the amount of unexpectedness of the cessation of reward, A(i) at the beginning of extinction was set to the reward probability that was learned during acquisition. Because large β (0.2) in Pan et al.’s model caused large fluctuations during the acquisition of probabilistic rewards–which should not have been apparent in the acquisition of deterministic rewards in their simulation–we reduced β to 0.04. Since small α (0.005) in their model required hundreds of acquisition trials, we increased it to 0.08 so that the number of acquisition trials in our model simulation is comparable to that in our empirical data (Fig. 2A). ψ^-^ and ψ^+^ were 0.999999 and 0.9, respectively. The length of each trial was 10 (arbitrary units). The CS and the reward were given at time points 3 and 8, respectively. The size of the eligibility trace decay factor λ was set to λ = 0.9 as in Pan et al. [20]. Acquisition sessions for simulations were constructed by combining the acquisition sessions that were actually used in our behavioral experiments. We assumed that extinction was completed when the size of the prediction error at the time of CS was below 0.12. We found that after these modifications, the model could produce spontaneous recovery and fast relearning after extinction as in the original model of Pan et al. (2008). We modeled the effect of dopamine inactivating drugs by modifying the size of the prediction error as follows.
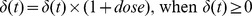



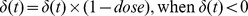



In contrast, drug-induced dopamine neuron activation was implemented as follows.
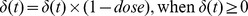





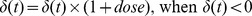
where 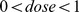 is the constant that represents the amount of the drug.

## Results

### Learned Value and Arousal may Influence the Shape of the Rate of Extinction vs Reward Probability Curve

Previous studies have found that, after extensive learning, the rate of the extinction of probabilistic rewards decreases as the reward probability increases (e.g. PREE) whereas, after moderate amount of learning, the rate of extinction follows an inverted U-shaped function of the reward probability [9]. In the latter case, the subject might not have learned enough to distinguish different probabilities of reward. Moreover, the difference in the number of rewards, instead of the reward probability itself, might have caused the inverted U-shape. Before addressing the effect of learned values and arousal on the extinction of probabilistic rewards, we proceeded to exclude these possibilities.

The rate of extinction was measured as the number of trials until extinction. Two-way RMANOVA indicated a statistically significant effect of the probability of reward on the rate of extinction (p = 0.005) (Fig. 2A). In trial-constant experiments, the extinction of 50% reward was significantly slower than that of 25% and 100% rewards (p = 0.020 and 0.041, respectively; n = 10; Fisher LSD method). In reward-constant experiments, the extinction of 50% reward was significantly slower than that of 25% and 75% rewards (p = 0.003 and 0.036, respectively). The effect of different experiments were not statistically significant (p = 0.163). There was no significant interaction between the reward probability and experiment type (p = 0.714). These results indicated that the reward probability itself, not the number of reward delivery, resulted in the inverted U-shape.

Our behavioral data include individual differences that do not exist in our model simulations. To ease qualitative comparison between our simulation results and empirical data, we normalized the number of trials until extinction (see methods) and analyzed again (Fig. 2B). Statistical tests on the normalized data gave similar results. The probability of reward had a statistically significant effect on the rate of extinction (p = 0.003; Two-way RMANOVA) (Fig. 2B). In trial-constant experiments, the extinction of 50% reward was significantly slower than that of 25% and 100% rewards (p = 0.019 and 0.050, respectively; n = 10; Fisher LSD method). In reward-constant experiments, the extinction of 50% reward was significantly slower than that of 25%, 75% and 100% rewards (p<0.001, p = 0.014 and 0.021, respectively). The effect of different experiments were not statistically significant (p = 0.222) and there was no significant interaction between the reward probability and experiment type (p = 0.499).

To confirm that the animals learned the probabilities of reward within the acquisition phase in both types of experiments, we analyzed the normalized reaction times during the acquisition phase of the trial-constant experiment (see methods for normalization; Fig. 2C). At the beginning of the acquisition phase, reaction times did not differ between the different reward probability conditions. However, by the end of the acquisition phase, the animals responded faster to rewards with higher probabilities. The reaction time was significantly shorter when the probability of reward was 50%, 75% and 100% than when the reward probability was 25% (p = 0.016, 0.003 and 0.002, respectively). Although not statistically significant, the average of reaction times at the end of the acquisition phase of 75% and 100% rewards were shorter than that of 50% reward. There was a significant effect of the reward probability on reaction times (p = 0.022; two-way RMANOVA). The effect of learning and interaction of learning and the reward probability were not statistically significant (p = 0.067 and 0.185, respectively). This result suggests that the animals learned different reward probabilities within the acquisition phase and that insufficient learning did not account for the inverted U-shape nature of the curves.

### Adding Arousal to a Temporal Difference Model Enabled the Simulation of the Inverted U-shape

The dependence of the pattern of extinction on the amount of learning indicates the importance of value learning in the extinction of probabilistic rewards. In addition, PREE suggests that arousal is a key factor in the extinction of probabilistic rewards. Previous studies have implicated midbrain dopamine neurons in both arousal and reinforcement learning [11], [17], [18], [22], [23]. Using model simulations, we investigated the interplay between value learning and arousal in the extinction of probabilistic rewards in terms of known activity pattern of dopamine neurons. A previous model related the phasic firing of dopamine neurons to the prediction error generated by temporal difference learning [20], [24], [25], [26]. This model successfully accounted for dopaminergic activity during acquisition and extinction of deterministic rewards and reproduced key features of extinction such as spontaneous recovery and fast re-learning [20], [26]. The model, however, did not take into account arousal and was not tested with probabilistic rewards.

**Figure 3:**
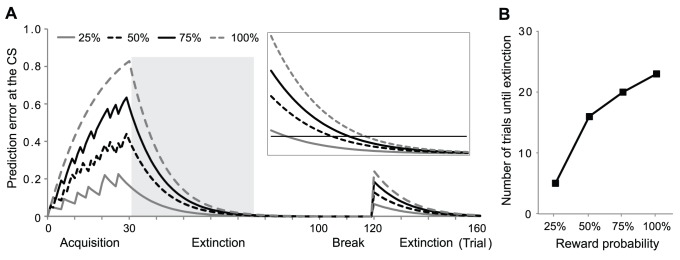
Simulation of the extinction of probabilistic rewards. **A:** Prediction error at the time of the CS during conditioning and extinction. The inset on the right is a horizontally enlarged view of the grey region. The black horizontal line in the inset indicates the point where the prediction error meets the criterion for extinction (0.12). The first 30 trials constitute the acquisition phase, the following 70 trials were the first extinction phase, the following 20 trials were the break (see methods) and the last 40 trials were the second extinction phase which shows spontaneous recovery. (λ = 0.9, α = 0.08, β = 0.04, ψ+ = 0.999999, ψ− = 0.9. **B:** Number of trials until the prediction error decays to the extinction criterion in the first extinction phase. The extinction of high reward probability is always slower than that of low reward probability.

We simulated the role of arousal in the extinction of probabilistic rewards (Fig. 3). In figure 3A, the levels of acquisition and extinction are shown as the size of the prediction error for a given conditioned stimulus (CS). 100% reward has a greater value than 50% reward. At the beginning of extinction (grey box), the magnitude of the prediction error of 100% reward probability is larger than that of 50%. However, the rate of decrease of the prediction error for 100% reward probability is not fast enough for its curve to cross the curve of the prediction error for 50% (Fig. 3A, inset). As a result, the extinction of 100% reward probability takes longer than the extinction of 50%. Without arousal, the extinction of high probability reward is slower than that of low probability rewards (Fig. 3B).

**Figure 4:**
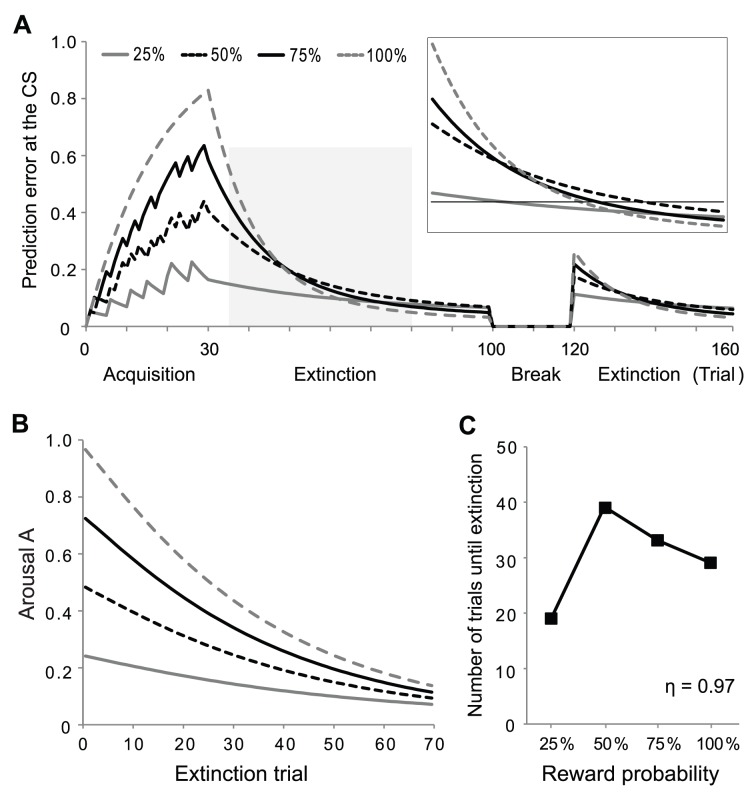
Simulation of the extinction of probabilistic rewards with arousal. **A:** Prediction error at the time of the CS during conditioning and extinction. All notations and values of parameters are the same as in Figure 3A. The decay factor of arousal η is 0.97. **B:** Arousal during the extinction phase. Arousal A changes more slowly than the prediction error. **C:** Number of trials until the prediction error decays to the extinction criterion in the first extinction phase. The model with arousal reproduced the inverted-U shape of the extinction probability curve seen experimentally.

Because the reliability of the prediction of reward delivery increases with the reward probability, the level of arousal at the beginning of extinction should also increase with the reward probability. Since arousal controls the learning rate, it hastens the extinction of higher reward probability but slows the extinction of lower reward probability. To reproduce the inverted U-shape, the curves of the prediction error for higher reward probability should cross those of lower reward probability during extinction (compare insets of Fig. 3A and Fig. 4A). For this to occur, the size difference between the arousal A of different probability should be maintained for a long enough period (Fig. 4B). We achieved this by introducing arousal in the extinction phase of the model by Pan et al. [20] and by setting the decay factor of arousal η to large values (Fig. 4B,C, see methods).

**Figure 5:**
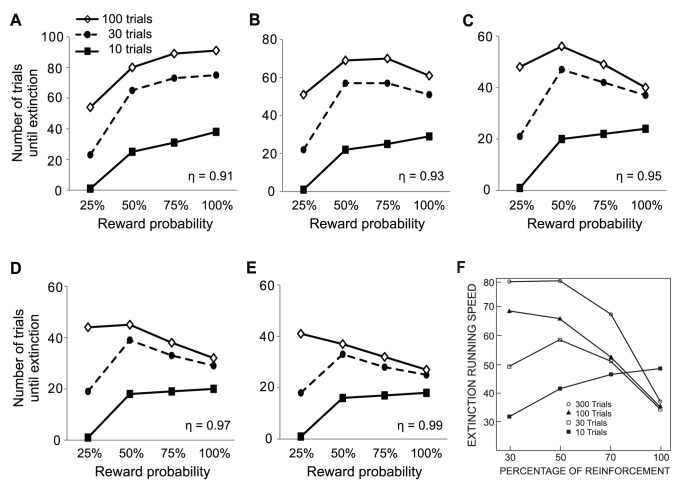
The amount of learning and the change rate of arousal affect the rate of extinction. **A–E:** Number of trials until extinction when the number of acquisition trials is 10, 30 or 100. The size of the decay factor of arousal η is 0.91 in **A**, 0.93 in **B**, 0.95 in **C**, 0.97 in **D**, and 0.99 in **E**. The values of all other parameters are the same as Figure 4A. **F:** Experimental data. Figure adapted from Bacon 1962 [9] with permission.

The plots of figure 5 A–E show the rate of extinction when the number of acquisition trials is 10, 30 or 100, respectively. These plots have increasing values of the decay factor from 0.91 to 0.99. With 30 acquisition trials, the inverted-U shape was reproduced with the decay factor η greater than 0.91 (Fig. 5B–E). Because the arousal and the prediction error continuously decayed during extinction, the curves of the prediction error became almost horizontal and parallel to one another by the end of extinction (Fig. 4A, B). Thus, for PREE to appear, large arousal should hasten the extinction of 50%, 75% and 100% reward probability during early to mid extinction so that the trajectories of the prediction error at the CSs of 50%, 75%, and 100% reward probabilities cross that of 25%. A slow decay of arousal (e.g. η = 0.99) gives a long temporal window for arousal to control the learning rate. As a result, the value of high probability rewards extinguishes more quickly than the value of low probability rewards, which causes PREE (Fig. 5E). In contrast, a fast decay of arousal (e.g. η = 0.91) leads to fast extinction of rewards with low values (low probability rewards in this case), resulting in a linear shape of the extinction curve (Fig. 5A). When the rate of decay of arousal is intermediate (e.g. η = 0.95), the extinction curve becomes a mixture of PREE and a linear function (Fig. 5C). In other words, PREE occurs only when the prior belief (and associated arousal) is persistent and fades very slowly whereas the inverted-U shape curve occurs with a faster decay of the prior belief. Experimentally, it is likely that the prior belief has been firmly established during extensive training which has been found to be important for PREE ([9] and see below) whereas the prior belief is less strong after intermediate amount of learning which has been found to be needed for the inverted-U. Significant portions of reward value are extinguished during early to mid extinction when both the prediction error and the arousal are large. Thus, as the decay factor of arousal η grew, extinction tended to occur faster.

As the number of acquisition trials increases, the learned reward value that has to be extinguished during extinction grows. As a result, extinction tended to become slower with a larger number of acquisition trials. Sufficient learning of the value of 25% reward gave more time for the curves of the prediction error for 50%, 75%, and 100% reward probabilities to cross that for 25% reward probability before extinction. Therefore, as the number of acquisition trials increases, the rate of extinction changed from a linear function, to an inverted-U shaped curve, and to an inversely proportional function of the reward probability (Fig. 5D–E). PREE only appears after extensive training. The trajectories of the prediction error at the time of the CS reached a plateau after around 90 acquisition trials (not shown). When the number of acquisition trial was 100, PREE was reproduced with the decay factor η greater than 0.97 (Fig. 5E). Thus, the amount of learning affects the shape of the extinction curve by determining the magnitude of reward value to be extinguished as well as by influencing the persistency of the prior belief. In all cases, our model reproduced spontaneous recovery and fast re-learning as the model by Pan et al. [20]. Our model with a decay factor η ≥0.97 (Fig. 5D, E), simulated well the effect of the amount of learning on the rate of extinction measured experimentally (Fig. 5F) [9].

### Model Prediction of the Effects of Enhancing and Reducing the Dopaminergic Prediction Error Signal

In our model, the prediction error is the key element regulating reward learning and arousal. Previous studies have indicated that dopamine neurons encode the prediction error [11], [24]. To further validate our model, we simulated the effect of enhancing and reducing the magnitude of the prediction error on the shape of the extinction curves and then tested the model predictions experimentally. Because phasic inhibition of dopamine is upper-bounded by the level of tonic firing, we implemented the reduction of the prediction error as negatively shifting the prediction error in a multiplicative way whereas enhancing the prediction error was implemented in the opposite way (see methods).

**Figure 6:**
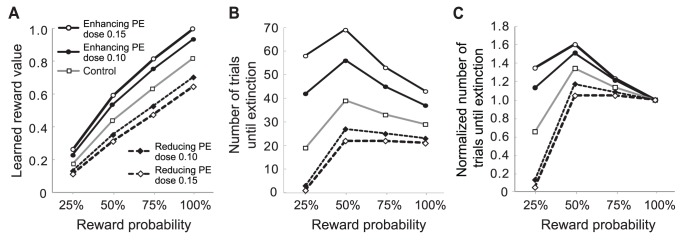
Model predictions of the effects of enhancing and reducing the magnitude of the prediction error. **A:** Effect of enhancing and reducing the magnitude of the prediction error on learned reward value at the end of the acquisition phase. The number of acquisition trials was 30 and the decay factor of arousal η was 0.97. **B:** Simulated effect of drugs enhancing/reducing the prediction error on the rate of extinction. **C:** Number of trials until extinction in B was normalized to the extinction of 100% reward probability in the same drug/dose condition. The normalization made it easier to compare the effect of drugs enhancing/reducing the prediction error on the shape of the extinction-probability curve.

Our model predicted that drugs that would reduce the size of the dopaminergic prediction error signal would result in an underestimation of reward value whereas drugs that would enhance the size of the dopaminergic prediction error signal would cause an overestimation (Fig. 6A). Reward underestimation and overestimation were further found to hasten and slow down extinction, respectively (Fig. 6B). This is because the amount of reward value that has to be extinguished is increased by overestimation but decreased by underestimation. Since low reward probabilities give only a small amount of reward value that has to be extinguished, the extinction of low probability rewards is particularly sensitive to the magnitude of learned value. Thus, the effect of enhancing and reducing the size of the prediction error on the rate of extinction was more prominent in low probabilities than on higher ones.

Our model also predicted that reducing the prediction error would flatten the shape of the function of the extinction rate and reward probability whereas enhancing the prediction error would bend it more (Fig. 6C). In the model, the overestimation from enhancing the prediction error increased the difference in the negative prediction error between high probability and low probability rewards during early extinction. Therefore, the ratio of the rate of extinction of high reward probability to that of low reward probability is higher when the prediction error is enhanced than when the prediction error is reduced. This resulted in the flatter shape of the function with reduced prediction error (Fig. 6C).

### Pharmacological Experiments in Rats Confirmed the Model Predictions

We tested the prediction of our model by pharmacologically activating and inactivating dopamine neurons of the ventral tegmental area (VTA). Because the tonic firing rate is smaller than the phasic firing rate, a drug that raises the overall firing of dopamine neurons would reduce the contrast between tonic and phasic firings. In addition, presynaptic dopamine D2 autoreceptors are activated by the tonic level of extracellular dopamine and inhibit dopamine release [27], [28]. Therefore, the infusion of such a drug into the midbrain would attenuate the positive prediction error. On the other hand, microinjection of this drug would enhance the magnitude of the negative prediction error because phasic inhibition of dopamine is upper-bounded by the level of tonic firing. A drug that suppresses the overall firing of dopamine neurons would have the opposite effects. Thus, we used a dopamine inactivating drug to enhance the magnitude of the prediction error while a dopamine activating drug was injected to reduce the size of the prediction error.

**Figure 7:**
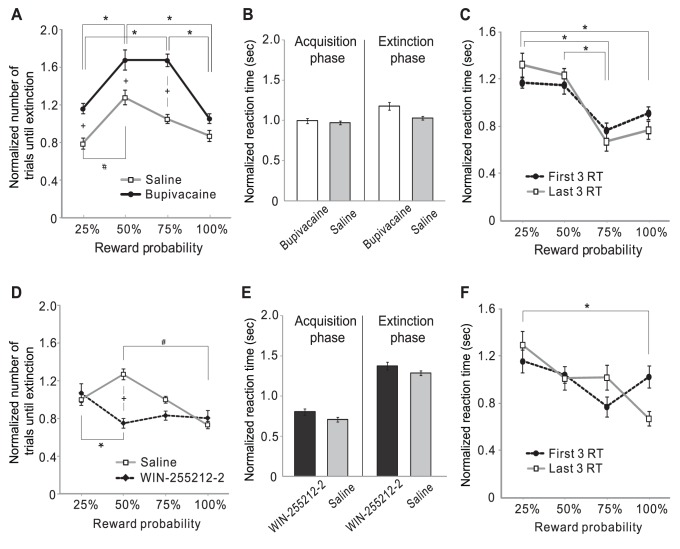
Effects of bupivacaine and WIN 55212-2 microinfusions into the VTA on extinction. Effect of bupivacaine (**A**) and WIN 55212-2 (**D**) on the rate of extinction (*, # and +: p<0.05 within the saline condition, within the bupivacaine/WIN 55212-2 conditions, and between the saline and bupivacaine/WIN 55212-2 conditions, respectively). Effect of bupivacaine (**B**) and WIN 55212-2 (**E**) on reaction times. Within the same phase, there was no significant difference between the drug conditions. First and last three reaction times during the acquisition phase in the bupivacaine (**C**) and WIN 55212-2 (**F**) conditions (*: p<0.05 within the last three reaction times).

To suppress dopaminergic activity, we microinjected bupivacaine into the VTA just before the acquisition phase. Two-way RMANOVA revealed statistically significant effects of the reward probability and drugs (p = 0.007 and p<0.001, respectively; n = 10). As predicted, bupivacaine slowed extinction and bent the shape of the extinction-probability curve (Fig. 7A, black curve). In the case of 25%, 50% and 75% reward probabilities, the rate of extinction was significantly slower in the bupivacaine condition than in the saline condition (p = 0.035, p = 0.025 and p<0.001, respectively; n = 10; Fisher LSD method). After bupivacaine infusion, the extinction of 50% and 75% reward probability was significantly slower than the extinction of 25% and 100% (compared to 50%: p = 0.006 and 0.020, respectively; compared to 75%: p = 0.006 and 0.021). There was no significant interaction between the effects of the drug and reward probability on the rate of extinction (p = 0.452). Taken together, these experimental results confirm our model prediction of the effect of enhancing the size of the dopaminergic prediction error signal. (Fig. 6B).

To increase dopaminergic activity, we used WIN-255212-2 (WIN2), a cannabinoid CB1 receptor agonist (Fig. 7D). Consistent with our assumption for the simulation, previous studies have found that WIN2 causes almost twice greater percent increase in tonic firing than in burst firing [29] and decreases evoked dopamine release [30]. WIN2 generally lowered and flattened the inverted U-shape of the extinction-probability curves. A statistically significant influence of the interaction between the effects of the drug and reward probability on the rate of extinction was found (p = 0.038; n = 11; two-way RMANOVA). The rate of extinction at 50% reward probability significantly increased in the WIN2 condition compared to saline (p = 0.007). This result is similar to that of our model simulations for the effect of the reduction of the prediction error with the simulated dose of 0.1, except for the extinction at 25% probability (Fig. 6B). In our model prediction with the dose of 0.1, 25% reward was extinguished after three trials, which was not the case in our experimental settings. During the acquisition phase of 25% reward probability, a pellet was provided every 3–5 trials. The fact that the rat did not stop approaching the tweezers to get food during the acquisition phase of 25% reward suggests that the animal was willing to make an effort to get pellets from the tweezers despite 4 consecutive unrewarded trials. One possible explanation is that the value of the tweezers was higher than zero at the beginning of the acquisition phase due to pre-training. Because it was difficult to know the exact value of the tweezers at the beginning of the acquisition phase, we set the value of the CS at the beginning of the acquisition phase to zero in our simulations rather than a higher arbitrary value. This difference could have caused the difference between the model prediction and experimental results. The extinction rate after WIN2 microinjection in our results might be the possible maximum rate in our experimental settings.

There was no statistically significant difference in the reaction times between saline and WIN-255212-2 microinjections (p = 0.266; n = 11; two-way ANOVA) or between saline and bupivacaine (p = 0.208; n = 7) (Fig. 7B, D). Thus, the effect of bupivacaine and WIN2 on the rate of extinction was not due to different levels of locomotor activity after the drug injection. To see if the drugs impaired the learning of reward probability during the acquisition phase, we compared the first three reaction times and the last three reaction times under each drug condition (Fig. 7C, E). In both bupivacaine and WIN2 conditions, the animals responded significantly faster when the reward was delivered with 100% probability than with 25% probability (p = 0.023 and p = 0.045, respectively). Although statistically not significant, the animals showed a tendency to respond faster to high reward probability. These results suggest that drug doses we used did not impair learning.

## Discussion

Using rat experiments and model simulations, we investigated the role of dopamine in the extinction of probabilistic rewards. We showed that arousal allowed the prior belief about the probability of reward to have lasting effects on the rate of extinction and hastened the extinction of high reward probabilities when compared to low reward probabilities. We also demonstrated that, by signaling the reward prediction error, dopamine determined the learned reward value that had to be extinguished during extinction and modulated an arousal signal that controlled the learning rate. Our model reproduced and explained the dependence of the rate of extinction on the reward probability on the amount of learning.

In our model, the effect of dopamine inactivation was implemented as positively shifting the prediction error in a multiplicative way whereas the effect of dopamine activation was implemented in the opposite way [31]. Consistent with this implementation, previous studies have found that the CB1 agonist WIN-255212-2 that we used to enhance dopamine firing caused almost twice greater percent increase in tonic firing than burst firing [29] and decreased evoked dopamine release [30]. Our implementation is compatible with prior studies in which dopaminergic modulation was implemented as a positive shift of the prediction error in response to activating drugs (e.g. L-DOPA) in Parkinson’s disease [32]. Because Parkinson’s disease involves dopamine neuron degeneration, the level of extracellular dopamine should be largely driven by the level of tonic firing in this disease [33]. Our simulation results revealed that scaling up the prediction error raised the difference in the value of rewards with distinct probabilities through overestimation and slowed extinction (Fig. 6AB). This result is in line with recent studies which suggested that low level of tonic activity facilitated exploitation but reduced exploration by enhancing the contrast between these different behavioral options [34].

In the present study, microinfusion of WIN-255212-2 that enhanced tonic firing of dopamine neurons hastened extinction. This result is seemingly in contradiction with previous studies that have linked tonic dopamine release with motivation [35]. These studies have suggested that high level of tonic dopamine encodes reward-rich environment where opportunity cost per unit time is high and enhances the vigor of the animal. Unlike phasic dopamine signaling, tonic dopamine firing changes at time scales that seem too slow to control specific behaviors. Whereas the role of phasic dopamine has been emphasized in reinforcing the conditioned response, high level of tonic firing has been linked with distraction in working memory paradigms [36] and increased exploration [34]. High level of tonic dopamine during extinction would raise the perceived opportunity cost per unit time while the value of the conditioned response decays. Thus, the animal would quickly give up the conditioned response and could be easily distracted by task-irrelevant stimuli, in turn causing faster extinction. Anecdotally, in some animals, we did observe an increase in locomotor activity and an increase in distraction after WIN-255212-2 injection.

Unlike *de novo* acquisition of the association between the CS and US, extinction involves changes in prior beliefs about the CS-US relationship. The well known phenomena of spontaneous recovery and reinstatement [1] suggest that the effect of prior beliefs on later learning is strong and persistent and emphasize the distinction between *de novo* acquisition of a belief and changes of prior beliefs. Previous studies have observed PREE even when a block in which the reward was delivered with 100% probability was inserted between acquisition and extinction of probabilistic rewards [37], [38]. It suggests that the inserted 100% probability block did not erase the original prior belief about the prediction probability of the CS and that the arousal elicited by deviations from the original prior belief did have lasting effects on extinction to generate PREE. It also revealed that PREE does not result from the greater difficulty in detecting the cessation of probabilistic rewards compared to deterministic rewards. Indeed, adding the arousal signal to the acquisition phase in our model failed to reproduce the inverted-U shape of the extinction-probability curve and PREE. Whereas arousal had to change very slowly during extinction to simulate the inverted-U shape and PREE, acquisition did not occur if arousal was not quickly updated by the prediction error. This implies that the slowly changing nature of arousal may help prior beliefs to have lasting influence on later learning by controlling the learning rate [3], [4], [39].

In a complex and changing environment, the observed probability of a reward may deviate from the animal’s prior belief (deviation represented as the prediction error). In addition, once changed reward probability may go back to its original value for unknown reasons (e.g. re-conditioning after extinction). Thus, building up a repertoire of beliefs should be a better strategy than maintaining and updating a single belief [40], [41]. For an animal with limited resources in the noisy real world, having a repertoire with several beliefs with meaningful differences should be more adaptive than having a repertoire with a large number of beliefs with minute distinctions. Therefore adding a new belief only when observations *significantly and consistently* deviate from existing prior beliefs should be appropriate [3], [5], [42]. Compared to the prediction error that is sensitive to small deviations from the prior belief and promptly updates trial by trial [15], [43], arousal is elicited by relatively large deviations and changes more slowly. It may allow arousal to sustain high learning rates until the majority of a new belief is formed when substantial deviations from prior beliefs are observed. Supporting this notion, previous studies have found that a significant deviation from prior belief raises the learning rate and that the learning rate decays more slowly than the prediction error [4], [15], [39]. Our simulations also indicated that the time constant of arousal should be large to reproduce the inverted-U shape and PREE.

However, the time constant and the level of dependence on the prediction error of arousal may vary depending on the extent to which prior beliefs are different from the new belief. In reversal learning where the content of the prior belief is simply flipped over and potentially different brain regions are involved in [44], [45], the fitted time constant was smaller than ours [15]. Further research should be conducted to understand how different types of alterations to prior beliefs may change the time constant and the level of dependence on the prediction error of arousal.

Recent studies have pointed to the amygdala as a potential neural substrate for arousal that is elicited by significant deviation from prior beliefs. Activity in the amygdala has been found to correlate with the pattern of arousal during reversal learning and switching [15], [16]. Inactivation of the amygdala delays reward extinction and task switching, suggesting a role of the amygdala in controlling the learning rate when abrupt deviations from prior beliefs occur [16], [46]. Because dopamine neurons signal the prediction error, they can inform the amygdala of abrupt deviations from prior beliefs. Indeed, a recent study found that the arousal signal of the amygdala disappeared when midbrain dopamine neurons were degenerated with 6-OHDA [17]. The Pearce-Kaye-Hall model proposed that the prediction error affects the level of arousal that controls the learning rate [5],[47]. Recent empirical studies, compatible with the present study, found that neural activity of the amygdala and midbrain dopamine neurons fit the pattern of the prediction error and arousal predicted by this model [15], [16]. The properties of prior beliefs proposed above resemble those of hippocampal memory traces [40]. The characteristic circuit of the hippocampus suited for pattern completion and separation may support formation and retrieval of prior beliefs [42]. It is likely that the amygdala encodes arousal receiving the prediction error from dopamine neurons and assists the formation of a new belief in the hippocampus by sustaining high learning rate using slowly changing arousal signals. It would be worth comparing activity in the amygdala during the inverted-U or PREE and our model predictions of the arousal signal.

The idea that the level of deviation from the prior belief controls the rate of learning of a new belief is amenable to Bayesian probability modelling. However, when arousal was defined based on the likelihood of reward omission given the reward probability as prior in our model, arousal was updated too fast to reproduce the inverted-U shape (not shown). Although more sophisticated Bayesian models successfully simulated the extinction of probabilistic rewards [42], it is not straight-forward to identify which neural activity corresponds to what parameter in those models. We chose here to define arousal as in the Pearce-Kaye-Hall model which directly links the level of arousal to the prediction error of dopamine [5], [47]. Bayesian models have the advantage that they are more flexible and widely applicable compared to the Pearce-Kaye-Hall model and a Bayesian approach would be helpful for investigations on arousal in various types of learning [4], [39], [48], [49]. However, the present study suggests that simplified implementation of arousal as in the Pearce-Kaye-Hall model can provide insights and straight-forward links to potential neural correlates and may generate helpful predictions (e.g. the arousal signal in the amygala during PREE).

The original Pearce-Hall model and other related studies have considered arousal in terms of enhanced attention by uncertainty and predictiveness [50], [51]. We interpreted arousal more broadly as a surprise elicited by discrepancies from prior beliefs and assumed that more than one prior belief may exist regarding each stimulus. An advantage of this view of arousal is that it helps investigation of diverse aspects of learning that are difficult to be addressed with arousal as a function of predictiveness and/or uncertainty. For example, the effect of the level of firmness of the prior belief on learning (e.g. In our simulations, PREE for which extensive learning has been found to be important occurred only when the prior belief was persistent and faded very slowly whereas the inverted-U shape curve for which intermediate amount of learning has been found to be needed occurred with a faster decay of the prior belief) can be examined. The content of the prior belief can also be diversified, not being confined to predictiveness/uncertainty of the CS (e.g. learning under the influence of prejudice or prior instruction from authority). Moreover, this view of arousal can be useful in investigating the formation of multiple memory traces about a single stimulus and their relevant neural correlates (e.g. hippocampal pattern separation).

In the present study, low probability rewards had lower values than high probability rewards because the amount of reward delivered each time was the same in the two conditions. However, PREE has also been found to occur when the values of rewards with distinct probabilities were equal [8]. We observed that the model reproduced PREE when values of rewards with different probabilities were the same (not shown). This is because the low arousal of low reward probabilities slows the extinction, whereas high arousal of high reward probabilities hastens the extinction.

Gambles with low probability of winning and large amount of rewards for each winning should maintain low levels of arousal and have considerable reward values. A person who casually starts a gamble with small amount of betting money would experience a low arousal. If the bet is raised, the value of the gamble would increase. However, the low arousal of the gamble which changes slowly would prevent negative prediction errors in frequent losses from driving extinction [7]. Pathological gambling patients might tend to develop lower arousal of gambles or may tend to be reluctant to modify these values, once established [6].

Using rat experiments and model simulations, the present study suggested that arousal allowed the prior belief about the probability of reward to have lasting effects on the rate of extinction of probabilistic rewards and that the prediction error mediated by dopamine activity is involved in this process. Cases where the equations for arousal in our model can be directly applied are confined to reinforcement learning that involves changes in the probability of reward. However, our generalized interpretation of arousal as a surprise signal elicited by deviations from prior beliefs may easily be adapted to various forms of learning that involves changes in prior beliefs–such as set shifting, latent inhibition, learning under prejudice or prior instruction from authority [52], [53]. In addition, our model would help understand abnormal extinction in pathological gambling disorders.
